# Oncogenic Mutation BRAF V600E Changes Phenotypic Behavior of THLE-2 Liver Cells through Alteration of Gene Expression

**DOI:** 10.3390/ijms23031548

**Published:** 2022-01-28

**Authors:** Magdalena Śmiech, Paweł Leszczyński, Christopher Wardell, Piotr Poznański, Mariusz Pierzchała, Hiroaki Taniguchi

**Affiliations:** 1 Department of Experimental Embryology, Institute of Genetics and Animal Biotechnology, Polish Academy of Sciences, 05-552 Jastrzębiec, Poland; m.smiech@igbzpan.pl (M.Ś.); p.leszczynski@igbzpan.pl (P.L.); 2 Department of Biomedical Informatics, University of Arkansas for Medical Sciences, 4301 W Markham Steet, Little Rock, AR 72205, USA; cpwardell@uams.edu; 3 Department of Experimental Genomics, Institute of Genetics and Animal Biotechnology, Polish Academy of Sciences, 05-552 Jastrzębiec, Poland; p.poznanski@igbzpan.pl; 4 Department of Animal Genomics and Biodiversity, Institute of Genetics and Animal Biotechnology, Polish Academy of Sciences, 05-552 Jastrzębiec, Poland; m.pierzchala@igbzpan.pl

**Keywords:** BRAF mutation, liver cancer, hepatocellular carcinoma, MAPK/ERK

## Abstract

The accumulation of mutations in cancer driver genes, such as tumor suppressors or proto-oncogenes, affects cellular homeostasis. Disturbances in the mechanism controlling proliferation cause significant augmentation of cell growth and division due to the loss of sensitivity to the regulatory signals. Nowadays, an increasing number of cases of liver cancer are observed worldwide. Data provided by the International Cancer Genome Consortium (ICGC) have indicated many alterations within gene sequences, whose roles in tumor development are not well understood. A comprehensive analysis of liver cancer (virus-associated hepatocellular carcinoma) samples has identified new and rare mutations in B-Raf proto-oncogene (*BRAF*) in Japanese HCC patients, as well as BRAF V600E mutations in French HCC patients. However, their function in liver cancer has never been investigated. Here, using functional analysis and next generation sequencing, we demonstrate the tumorigenic effect of BRAF V600E on hepatocytes (THLE-2 cell line). Moreover, we identified genes such as *BMP6, CXCL11, IL1B, TBX21, RSAD2, MMP10,* and *SERPIND1*, which are possibly regulated by the BRAF V600E-mediated, mitogen-activated protein kinases/extracellular signal-regulated kinases (MAPK/ERK) signaling pathway. Through several functional assays, we demonstrate that BRAF L537M, D594A, and E648G mutations alone are not pathogenic in liver cancer. The investigation of genome mutations and the determination of their impact on cellular processes and functions is crucial to unraveling the molecular mechanisms of liver cancer development.

## 1. Introduction

Nowadays, an increasing number of chronic liver diseases and cancer cases is observed and also has a high mortality rate, amounting to 2 million precocious deaths annually worldwide [1]. The most common malignant primary liver cancer is hepatocellular carcinoma (HCC), which is derived from hepatocytes. Approximately 90% of liver cancer refers to HCC [2]. The highest incidence of primary liver cancer is in Asia and Africa, whereas in Europe it is at a moderate level. Recently, a significant increment of liver cancer patients has been observed, especially in the USA and Europe [3,4]. Several main risk factors predispose patients to developing liver cancer, including the following: long-term viral infection [1], alcohol abuse [5], exposure to toxins, such as aflatoxin [6,7], and metabolic disorders, such as obesity, diabetes, and fatty liver disease [8,9]. These elements may lead to the accumulation of mutations in cells and to malignant hepatocyte transformation [10]. Several cancer driver mutations in the development of HCC are already distinguished in genes, e.g., telomerase reverse transcriptase (*TERT*) [11], AT-rich interaction domain 1A and 2 (*ARID1A* and *ARID2*) [12], catenin beta 1 (*CTNNB1*) [13,14], or tumor protein p53 (*TP53*) [15,16]. Comprehensive data provided by the International Cancer Genome Consortium (ICGC) [17] contain many mutated genes in HCC, including the B-Raf proto-oncogene, serine/threonine kinase (*BRAF*) gene.

BRAF belongs to the RAF family of serine/threonine protein kinases and is a crucial component of the mitogen-activated protein kinases/extracellular signal-regulated kinases (MAPK/ERK) signaling pathway [18,19], regulating a wide range of cellular processes, such as apoptosis, stress responses, proliferation, and differentiation [20]. Proper regulation of cell functions is crucial for maintaining the homeostasis between extracellular signals and internal response to stimuli. Over 20 years of studies have revealed that alterations in the MAPK/ERK pathway and aberrant signal transduction is a major trigger for the development of various cancer types [21]. Approximately 30% and 8% of all cancer types are related to *KRAS* and *BRAF* mutations, respectively [20]. The most common mutation in the *BRAF* gene is a transversion of thymidine (T) to adenosine (A) at nucleotide 1799. It results in the replacement of valine (V) with glutamic acid (E) in codon 600 (V600E) [22]. BRAF V600E mutations cause high kinase activation and constitutive signal transduction in a RAS-independent way [23], leading to an increased rate of cell proliferation and resistance to apoptosis [24]. Although mutations in BRAF may affect malignancies in several cancers, functional analysis of BRAF V600E and other mutations has not been reported in HCC.

Large-scale sequencing (data available on ICGC data portal) has identified various mutations associated with the pathogenesis of many cancer types [25]. Importantly, the analysis of 300 liver cancer tissues—268 HCC, 24 intrahepatic cholangiocarcinoma (ICC), 8 of both types (HCC/ICC)—has identified new BRAF gene mutations for primary liver cancer [26]. BRAF V600E and BRAF D594A are known mutations in various cancer types but their functional impact on liver cells is not defined. BRAF E648G and BRAF L537M are newly discovered mutations in HCC. Therefore, in this study, we assessed the tumorigenic effect of these mutations on THLE-2 cells. Moreover, changes in gene expression of THLE-2 cells by overexpressing these mutations were evaluated. Our study demonstrated a tumorigenic role of BRAF V600E mutation and identified possible target genes for BRAF V600E-mediated MAPK/ERK signaling pathway in THLE-2 liver cells.

## 2. Results

### 2.1. Selected BRAF Mutations Are Located in the Kinase Domain of BRAF

**Figure 1:**
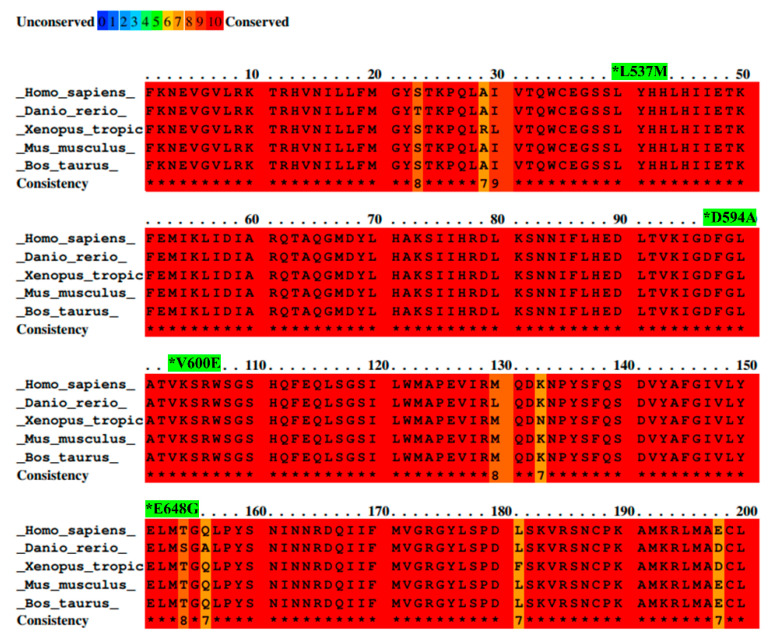
Alignment of BRAF amino acid sequences from different species with mutation sites indicated as *. Sequence alignment was performed between species—*Homo sapiens, Mus musculus, Bos taurus, Danio rerio*, and *Xenopus tropicalis*—by PRALINE software. Results are shown as a color-coded pattern. The scoring scheme ranges from 0 (for the least conserved alignment position) (in blue) up to 10 (for the most conserved alignment position) (in red). BRAF mutations selected for study have exhibited the highest conservation score between species.

BRAF L537M, D594A, V600E, and E648G mutations were identified by the ICGC in Japanese and French liver cancer patients and investigated to reveal their possible roles in liver cancer development. All details concerning these mutations are presented in Table 1. Additionally, the amino acid sequence of the part of the activation segment located in the BRAF kinase domain has exhibited a high conservation score among selected species. Moreover, the color scheme comparison presented in Figure 1 confirms that the locus containing the BRAF mutations (L537M, D594A, V600E, and E648G) is highly evolutionarily conserved across several organisms. This suggests the high importance of BRAF protein structure and emphasizes a key role of BRAF kinase in the regulation of cellular processes among organisms.

### 2.2. Effect of BRAF Mutations on Its Kinase Activity

**Figure 2:**
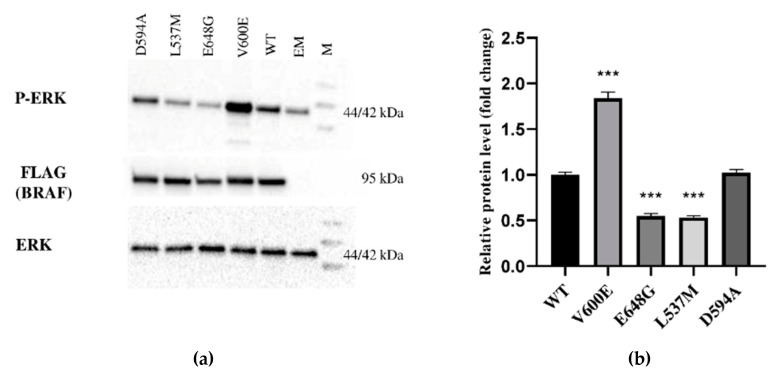
Protein expression measured by Western blot. (**a**) Expression of ERK-phosphorylated (P-ERK), FLAG (BRAF), and ERK proteins in THLE-2 cells overexpressed with p3XFLAG-CMV empty plasmid (EM), or plasmid containing BRAF WT (WT) and BRAF mutations (V600E, E648G, L537M, D594A). The density of each band was quantified by ImageJ software (**b**) The protein expression level was normalized to FLAG (BRAF) and presented as a fold change (±SD) of band intensity in the presented blot. The experiment was conducted in four independent replicates (*n* = 4) and analyzed using a one-way analysis of variance (ANOVA) with Bonferroni correction, *** *p*  <  0.001. The expression of ERK proteins was equal for all samples. M—protein marker 10–245 kDa.

The expression of BRAF (FLAG), ERK, and P-ERK proteins were measured in THLE-2 cells overexpressed with p3XFLAG-CMV empty plasmid (EM) or plasmid containing BRAF WT (WT), BRAF V600E, E648G, L537M, or D594A. This study showed differences in signal transduction in the MAPK/ERK signaling pathway depending on BRAF mutation. BRAF as a kinase activates (via MEK) ERK by phosphorylation. Here, we evaluated the impact of selected BRAF mutation on its kinase activity (Figure 2). As a result, we observed the augmentation of P-ERK level in THLE-2 cells with overexpression of BRAF V600E. Interestingly, significant down-regulation of P-ERK was observed in BRAF E648G and BRAF L537M-overexpressed THLE-2 cells. No effect was found for THLE-2 cells overexpressed with BRAF D594A. All samples expressed comparable levels of ERK protein. β-actin expressions are presented in Figure S1.

### 2.3. Motility of THLE-2 Cells with BRAF Mutations

**Figure 3:**
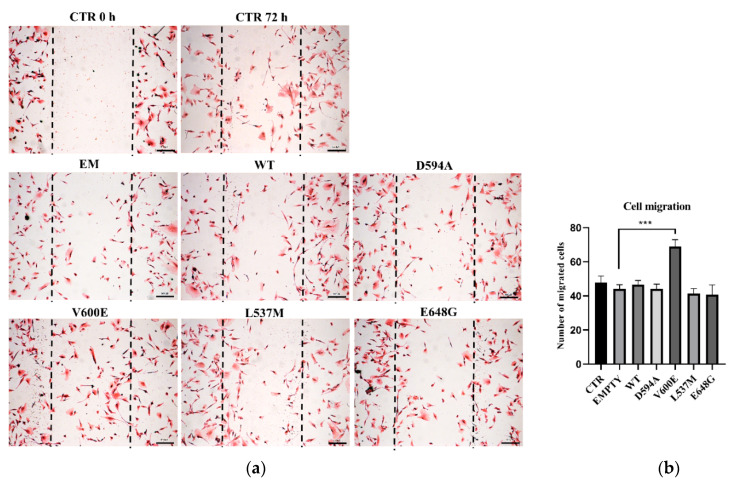
Cell migration assay on THLE-2 cells with overexpression of BRAF mutations. (**a**) Scratch assay picture of non-transfected (CTR) THLE-2 cells, transfected with BRAF wild type (WT), mutants (D594A, V600E, L537M, E648G), and empty plasmid (EM). Dark dashed lines indicate the trace of the wound front. Scale bar 200 μm. (**b**) Bar chart with the number of migrated cells (±SEM) for all experimental groups, *n* = 3, *** *p*  <  0.001. The statistical analysis was carried out using ANOVA with Bonferroni correction.

Next, we aimed to evaluate THLE-2 cells’ migration ability after overexpression of BRAF mutations (D594A, V600E, L537M, and E648G). The results demonstrate that cell migration was significantly augmented in BRAF V600E-overexpressed THLE-2 cells compared with control (BRAF WT) cells (Figure 3). The migration level of THLE-2 cells overexpressed with BRAF D594A, BRAF L537M, or BRAF E648G was comparable to control cells. No significant effect on cell migration was observed in THLE-2 cells transfected with backbone (empty) plasmid.

### 2.4. Effect of BRAF Mutations on THLE-2 Cells Proliferation

**Figure 4:**
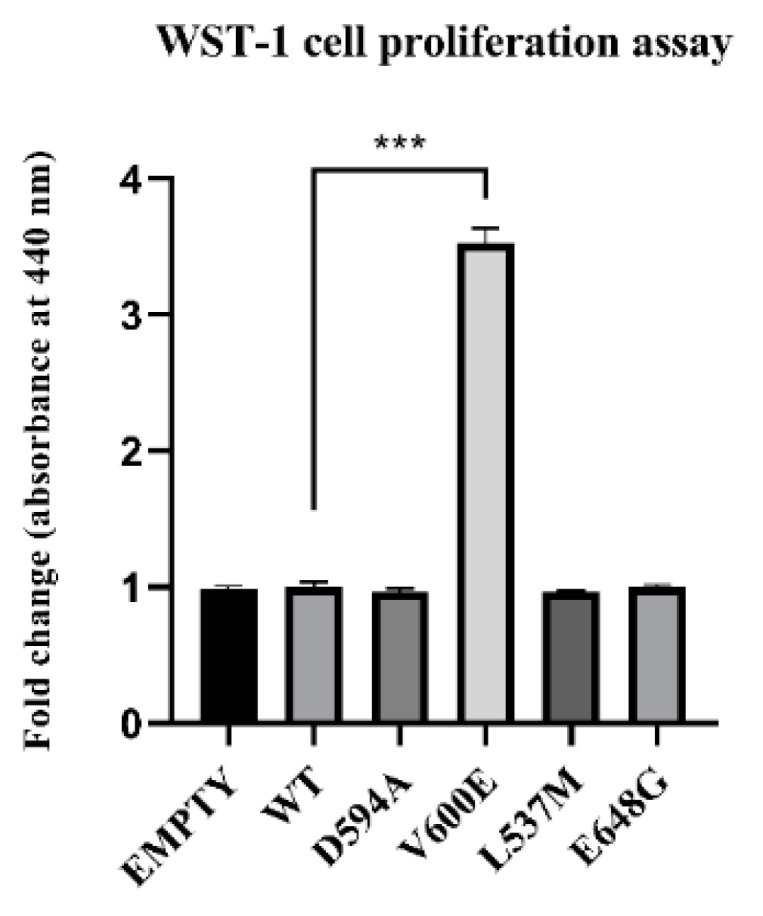
Effect of BRAF mutant overexpression in THLE-2 cells on cell proliferation. A statistically significant augmentation in hepatocyte proliferation, when compared with control, was observed in BRAF V600E-transfected cells. The results are presented as a fold change (±SEM) normalized to BRAF WT-transfected cells. The statistical analysis was carried out using ANOVA with Bonferroni correction, *n* = 3, *** *p*  <  0.001.

The role of BRAF mutations on THLE-2 cell proliferation was measured after 24 h from cell transfection. Consequently, BRAF V600E mutation significantly increased THLE-2 cell proliferation compared with control cells (BRAF WT). No significant effect was observed in cells with BRAF L537M, BRAF D594A, and BRAF E648G overexpression. Similarly, backbone p3XFLAG-CMV (empty plasmid) did not influence the cell proliferation rate (Figure 4).

### 2.5. BRAF V600E Mutation Influences on Cell Invasion

**Figure 5:**
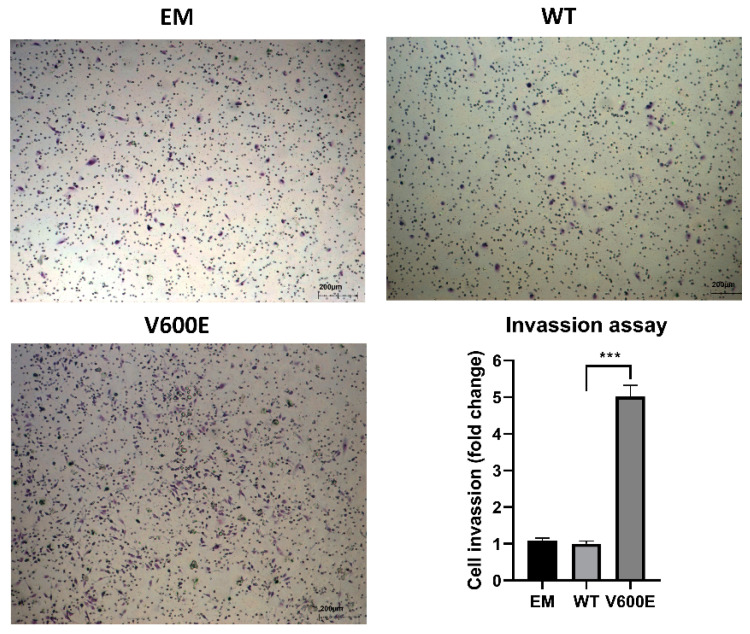
Effect of BRAF V600E overexpression on the THLE-2 cells invasion. THLE-2 cells exhibited augmented invasion capacity after overexpression of BRAF V600E compared with control cells. Quantitative data of invasion assay are presented as the mean ± SD, *** *p*  <  0.001. One-way ANOVA with Bonferroni corrections was applied for statistical analysis.

A trans-well migration assay was performed to assess whether BRAF V600E affects the hepatocytes (THLE-2 cells) invasiveness. As a result, THLE-2 cells overexpressed with BRAF V600E significantly enhanced invasion activity (Figure 5) compared with control cells (BRAF WT-transfected cells).

### 2.6. Analysis of Differentially Expressed Genes (DEGs) and Involved Pathways

**Figure 6:**
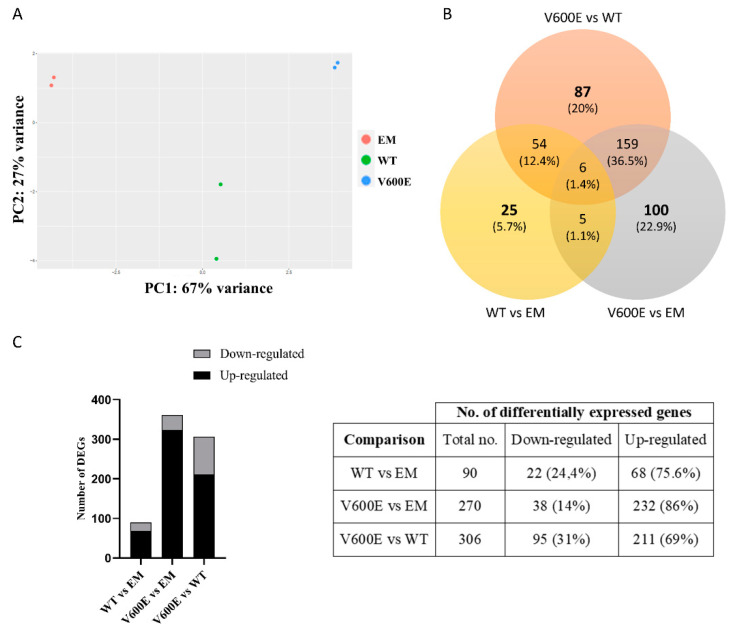
Principal component analysis (PCA) plot and the number of DEGs obtained from BGI RNA-Seq (transcriptome) sequencing. (**A**) The clustering of color-coded dots indicates high consistency between replicate samples. Dots refer to RNA samples extracted from (1) THLE-2 cells transfected with empty plasmid (EM) are marked in red, (2) BRAF WT-transfected THLE-2 cells (WT) are presented as green, (3) BRAF V600E-transfected THLE-2 cells (V600E) are marked in blue. (**B**) Venn diagram of DEGs. Three comparisons were analyzed, as follows: WT vs. EM, V600E vs. EM, V600E vs. WT. The overlapping part of the different circles indicates the number of DEGs common to respective groups. (**C**) The results indicated down-regulated and up-regulated genes in comparisons, as follows: WT vs. EM, V600E vs. EM, and V600E vs. WT. A *p*-value cut-off of 0.1 after Benjamini–Hochberg multiple-testing correction was used to fulfill the criteria of significance.

Transcriptome analysis indicated numerous DEGs and biological processes that may be regulated by BRAF V600E-mediated MAPK/ERK signaling pathway. In addition, principal component analysis (PCA) of the distribution of the analyzed samples indicated significant differences among compared groups and high similarity between duplicates within the group (Figure 6A).

**Figure 7:**
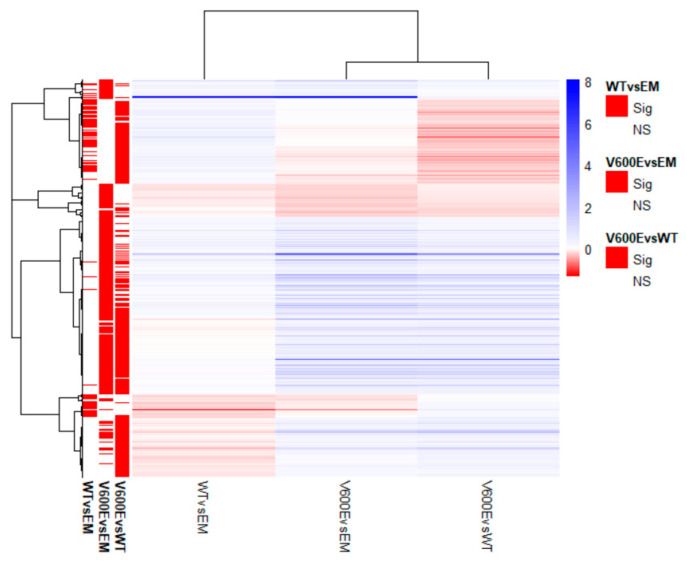
Heatmap of all significantly differentially expressed genes. Rows represent genes and each column is a contrast. The bars on the left display significantly expressed genes. In the main plot, the scale runs from red (negative fold change) to blue (positive fold change). The blue bar near the top of the plot is *BRAF* and *BRAFP1* (BRAF Pseudogene 1), where the fold change is highest compared with the empty plasmid-transfected cells.

Transcriptome analysis data revealed 436 significantly differentially expressed genes (DEGs) from all experimental variants. The score of 25 genes (5.7%) in WT vs. EM, 100 genes (22.9%) in V600E vs. EM, and 87 genes (20%) in V600E vs. WT comparisons were differentially expressed and unshared between other groups. The number of down-regulated or up-regulated genes distributed among the study groups are presented in the Venn diagram (Figure 6B). The obtained data indicated DEGs in three analyzed comparisons, as follows: 90 significantly DEGs in WT vs. EM (22 down-regulated and 68 up-regulated), 270 DEGs in V600E vs. EM (38 down-regulated and 232 up-regulated), and 306 DEGs in V600E vs. WT (95 down-regulated and 211 up-regulated) (Figure 6C). Moreover, DEGs were presented as a heatmap based on hierarchical clustering analysis (Figure 7). The columns represent WT vs. EM, V600E vs. EM, V600E vs. WT comparisons, where each row represent a gene. The most up- and down-regulated genes are presented in Table 2.

### 2.7. Validation of RNA-seq Data by qPCR

**Figure 8:**
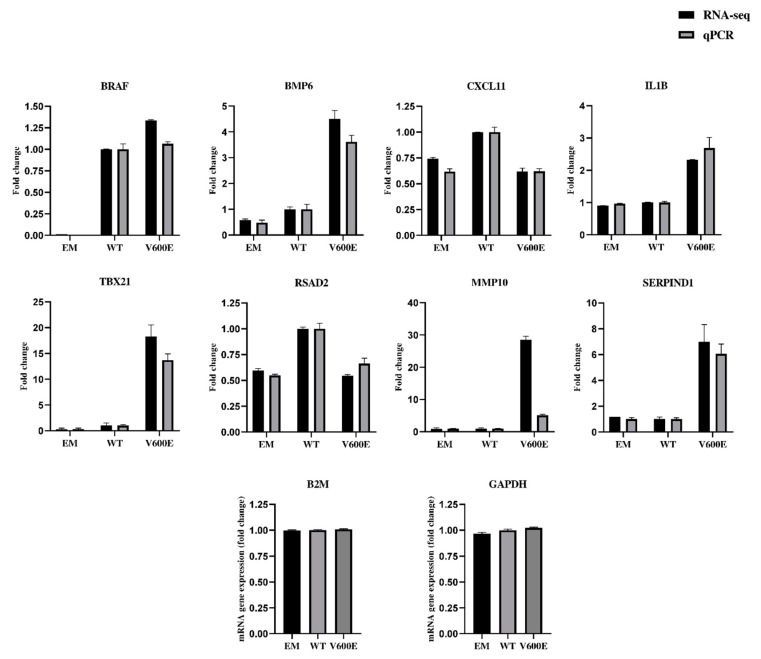
Validation of RNA-seq data by quantitative RT-qPCR (qPCR). The analysis was performed for 8 differentially expressed genes. Black bars represent results obtained from RNA-seq and gray bars indicate qPCR data. *B2M* was applied for the normalization of gene expression. All data are mean (±SEM), *n* = 2.

The qPCR results (presented in Figure 8 and Figure S3) confirmed the direction of changes in the genes expression. Genes were extracted for RNA-seq data validation, based on upregulation rate and their potential role in liver cancer development. Consistent data were collected for the following genes: *BRAF*, bone morphogenetic protein 6 (*BMP6*), C-X-C motif chemokine ligand 11 (*CXCL11*), interleukin 1 beta (*IL1B*), T-box transcription factor 21 (*TBX21*), radical S-adenosyl methionine domain containing 2 (*RSAD2*), and serpin family D member 1 (*SERPIND1*). Matrix metallopeptidase 10 (*MMP10*) was up-regulated in both RNA-seq and qPCR results but not by the same value. The expression of glyceraldehyde-3-phosphate dehydrogenase (*GAPDH*) and beta-2-microglobulin (*B2M*) were examined to identify optimal reference genes for the normalization of target gene expression data. *B2M* was used as an internal control for qPCR. The same RNA samples were used in qPCR and RNA-seq for assessment of gene expression level.

### 2.8. Canonical Pathway Analysis

**Figure 9:**
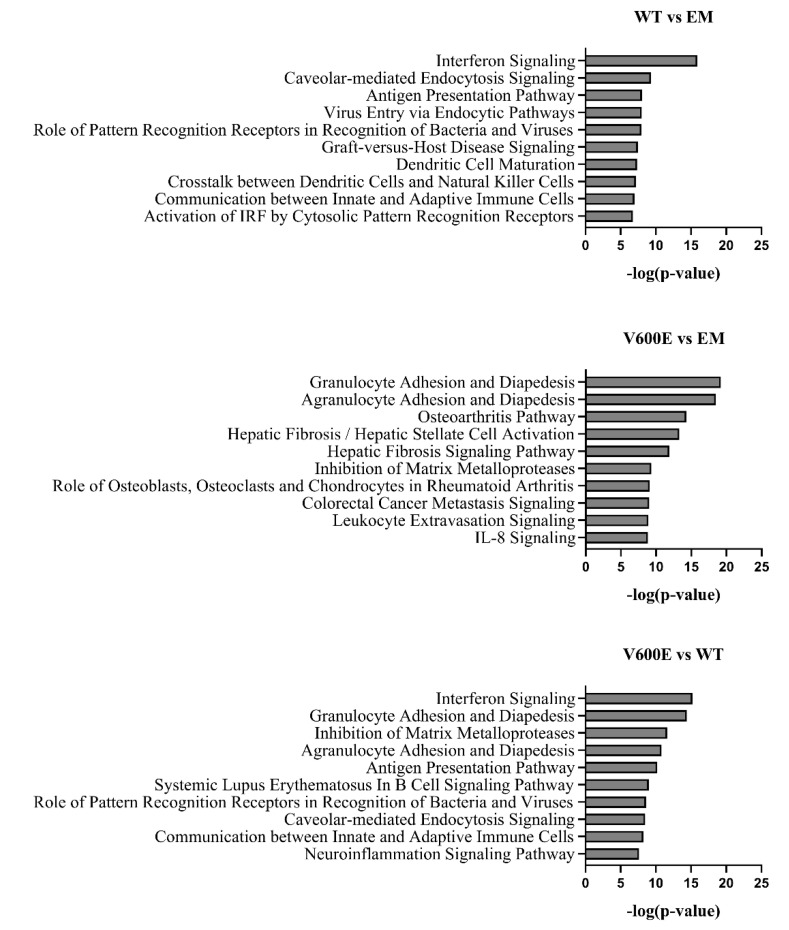
Top 10 significantly dysregulated canonical pathways. The bars present the various pathways in comparisons (WT vs. EM, V600E vs. EM, V600E vs. WT) based on a significant enrichment score (–log(*p*-value)).

The significant DEGs were assigned to canonical pathways to predict the impact of BRAF V600E mutation on cell processes and function. Results revealed numerous pathways with a significant enrichment score (–log(*p*-value)). Figure 9 represents the top 10 dysregulated pathways for all investigated groups. Considering the V600E vs. WT group comparison, the most impacted pathways include interferon signaling, granulocyte/agranulocyte adhesion and diapedesis, inhibition of matrix metalloproteases, and antigen presentation pathway, which are crucial in the regulation of immunity, inflammation, proliferation, and cell migration [27,28,29]. Moreover, in the V600E vs. EM comparison, some of the most dysregulated pathways were associated with the immune system and pathological processes in the liver, such as hepatic fibrosis, hepatic stellate cell activation, and the hepatic fibrosis signaling pathway.

### 2.9. Affected Pathway Analysis

**Figure 10:**
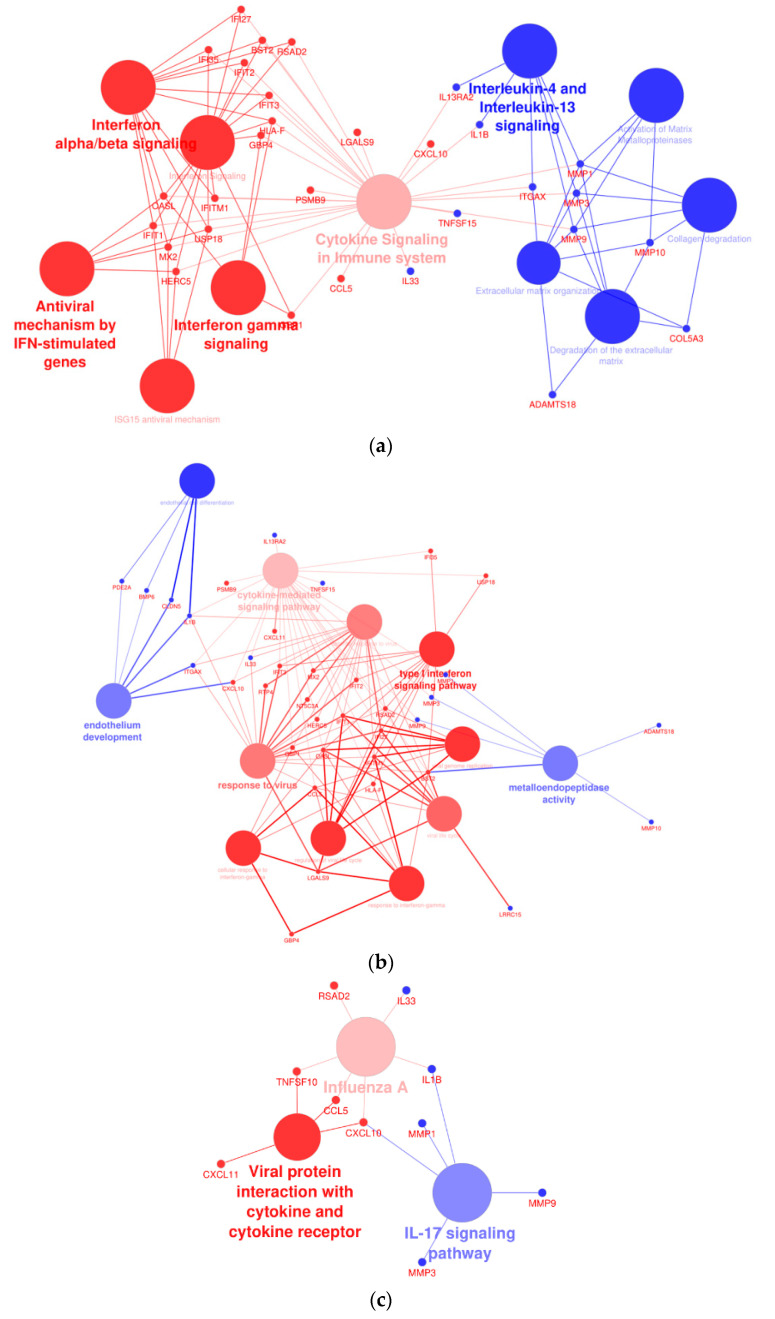
Cytoscape-ClueGo gene network interaction analysis, up- (blue) and down-regulated (red) genes. (**a**) Overexpression of BRAF V600E in THLE-2 cells influences the interferon- and metalloproteinase-dependent signaling pathways. (**b**) Analysis pointed out dysregulation of genes involved in the endothelium development and viral response. (**c**) An enhancement of IL-17 signaling pathway with attenuation of gene expression in response to viral infection and cytokine production.

A network analysis was performed to assess the interactions of dysregulated genes in THLE-2 cells with BRAF V600E overexpression compared with cells with overexpression of BRAF WT plasmid. The analysis revealed significantly altered processes, as follows: interferon signaling pathways, cytokine signaling, activation of metalloproteinase, endothelium development, or antiviral response (Figure 10).

### 2.10. Gene Expression Analysis in BRAF Mutant-Transfected THLE-2 Cells

**Figure 11:**
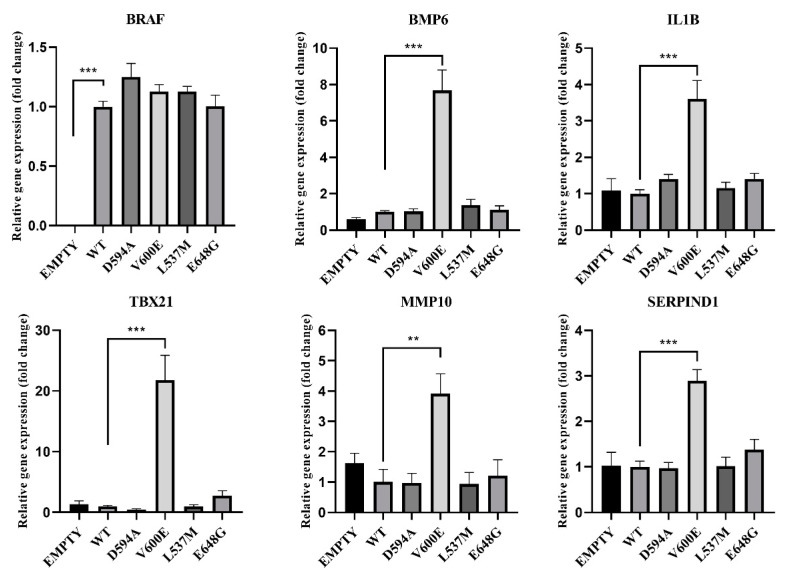
Effects of BRAF mutations on the selected gene expression. A significant upregulation (fold change) of *BMP6*, *IL1B*, *TBX21*, *MMP10*, and *SERPIND1* was seen in BRAF V600E-transfected THLE-2 cells. Results are presented as a fold change in contrast to the expression of BRAF WT-transfected cells (±SEM). *B2M* was subjected as a reference gene. The statistical analysis was performed using a one-way ANOVA with Bonferroni correction, *n* = 3, ** *p* < 0.01, *** *p*  <  0.001.

Based on the RNA-seq results, the gene expression was verified in THLE-2 cells transfected with BRAF mutants (D594A, V600E, L537M, E648G) for the following genes: *BRAF, BMP6, IL1B, TBX21, MMP10*, and *SERPIND1* (Figure 11). *BRAF* mRNA expression was nearly equal for all BRAF mutants and BRAF WT, which indicates that the transfection efficiency was at the same level for all experimental groups. Empty plasmid backbone did not influence BRAF expression in THLE-2 cells. Significantly increased expression level has been observed for *BMP6, IL1B, TBX21, MMP10,* and *SERPIND1* genes in BRAF V600E-overexpressed THLE-2 cells, whereas other BRAF mutants did not show changes in their regulation.

## 3. Discussion

Comprehensive data provided by the International Cancer Genome Consortium (ICGC) [17] have revealed thousands of alterations in the cancer genome. Further functional studies were therefore required to determine whether the found mutations are crucial in cancer development (cancer driver mutations) or are irrelevant and described as passenger mutations (not pathogenic) [30]. Interestingly, since mutations in the known cancer driver gene *BRAF* were found for the first time in a patient with liver cancer [25], we examined the effect of the mutations on hepatocytes. In the present study, we found V600E mutations to be pathogenic in HCC, but we did not find D594A, L537M, or E648G mutations alone to be pathogenic in HCC. More importantly, we identified several genes which are dysregulated by BRAF V600E in THLE-2 cells.

In the present study, high upregulation of ERK phosphorylation was observed in THLE-2 cells with BRAF V600E overexpression. This result supports the hypothesis that the BRAF V600E mutation strongly activates the MAPK/ERK signaling pathway in hepatocytes. Moreover, our results are consistent with studies performed by other research groups on different types of cancer caused by BRAF V600E mutation [31,32,33] and suggests that BRAF V600E mutation can be a risk factor for liver tumor development. Through RNA-seq analysis, a number of DEGs were identified by large-scale sequencing of the hepatocyte transcriptomes bearing BRAF V600E mutations. Since the MAPK/ERK signaling pathway is involved in regulation of many cellular processes, the identification of genes deregulated by BRAF V600E mutations are crucial to unravel the mechanisms of liver cancer development. ERK1/2 is located in the cytoplasm, but after activation it is transported to the nucleus where it stimulates the expression and activity of many key transcription factors [34,35,36] including proto-oncogenes such as *c-JUN, c-FOS, ELK-1*, or *c-MYC* [35]. Since it has been previously reported that MAPK/ERK signaling elicits similar gene deregulation (e.g., *BMP6, TBX21*, and *SERPIND1*) to our study [37,38,39], it is possible that BRAF V600E affects the downstream genes through MAPK/ERK-dependent transcription factors and dysregulates a series of gene expression in different cancer types. Further studies are needed to clarify this point. 

Interestingly, THLE-2 cells with BRAF V600E overexpression display a number of dysregulated genes, including serpin family D member 1 (*SERPIND1*)—a member of the serine proteinase inhibitor family synthesized by hepatocytes and macrophages [40,41]. Elevated levels of SERPIND1 are found in hepatocellular carcinoma, multiple myeloma, breast cancer, colorectal tumors, and other cancers [41,42]. In non-small-cell lung cancer (NSCLC) patients, increased SERPIND1 expression was associated with shorter overall survival rates [43], suggesting that *SERPIND1* may be a target gene of BRAF V600E. Nevertheless, how the BRAF V600E-MAPK/ERK axis regulates SERPIND1 at the molecular level is still unknown and requires identification of key transcription factors which may act as critical regulators of BRAF V600E-MAPK/ERK-dependent gene regulation in several types of cancer. The significantly dysregulated canonical pathways determined by DEGs (WT vs. V600E) revealed the most affected pathways in BRAF V600E-overexpressed THLE-2 cells. These include interferon signaling pathways, activation of metalloproteins, cytokine signaling, endothelium development, or antiviral response. Interferon (IFN) proteins are a group of factors produced by cells to activate protection against various disorders resulting from viral invasion or the development of cancer cells. IFNs can either contribute directly to the fight against cancer or indirectly activate and target the immune system to cancerously transformed cells [44]. IFN has been used for years as an adjunct to the treatment of melanoma [45]. The BRAF V600E mutation decreases the level of IFN-alpha receptor-1 (IFNAR1) and IFN-alpha-dependent signaling processes [46,47]. Additionally, it has been proposed that BRAF inhibitors support and enhance the anti-proliferative IFN-alpha effect on melanoma cells. Moreover, blocking the BRAF V600E-dependent signal results in increased expression of IFNAR1 [46,47]. The inhibitory effect of IFN-beta on the growth of hepatocellular cells has been shown in vitro and in vivo [48]. Therefore, it is assumed that the use of this therapy in the treatment of liver cancer patients carrying BRAF V600E somatic mutations may generate a similar therapeutic effect.

On the other hand, Cytoscape-ClueGo network analysis of BRAF V600E-overexpressed THLE-2 cells revealed dysregulation in the endothelial gene expression, such as bone morphogenetic protein 6 (*BMP6*). BMP6 is well known as a regulator of iron homeostasis, adipose, and bone tissue development [49,50]. It was showed that BMP6 overexpression promotes invasiveness and migration of prostate and breast cancer cells [51,52]. Finally, *Bmp6* deficiency in a mouse model of melanoma was linked with a large reduction in tumor progression [53]. BRAF V600E has a great influence on the mechanisms of tumor progression in various tissues. Constant activation of the MAPK/ERK pathway promotes cell migration, proliferation, and the tumor microenvironment [54]. Deregulated activation of the BRAF V600E-dependent MAPK/ERK effectors is best recognized in the mechanisms underlying melanoma genesis and the growth of papillary thyroid cancer [32,55,56]. A predominantly similar phenotype is caused by the V600E mutation, including enhanced invasion, proliferation, and metastasis [32,55,56]. Our studies showed a significant similarity of the influence of BRAF V600E on the characteristics of THLE-2 cells, which may indicate a strong invasive and migration effect of this mutation in the liver tissue. Our study also revealed that interleukin 1 beta (*IL1B*) gene upregulation is related to BRAF V600E overexpression in THLE-2 cells. The role of IL1B as a component of the inflammatory response is known in the context of regulation of various cellular processes, such as proliferation, migration, and apoptosis. However, many studies have indicated the significance of IL1B in tumors. IL1B promotes myeloid-derived suppressor cells (MDSC), angiogenesis, and endothelial cell activation and supports the immunosuppressive activity of tumor-associated macrophages (TAMs) [57]. Moreover, upregulation of IL1B has been reported in various solid tumors, including breast, colon, lung, melanoma, and others [58,59,60,61]. The study based on melanoma cell line and melanocytes proved that BRAF V600E mutation-induced transcription of *IL1B*. This effect could be blocked by vemurafenib (BRAF V600E inhibitor) treatment [62]. The role of matrix metallopeptidase 10 (*MMP10*) in cancer progression and metastasis has been studied in various cancer types and significantly increased expression of MMP10 was indicated in the cancer of skin, colon, lung, cervical tumors, and others [63,64,65,66]. A study using a murine HCC model has revealed that MMP10 was activated through the MAPK/ERK pathway and C-X-C chemokine receptor-4/stromal-derived factor-1 (CXCR4/SDF1) axis has contributed to hepatocellular cancer progression and metastasis [67]. Similar to these studies, we also observed upregulation of *MMP10* only in BRAF V600E-overexpressed THLE-2 cells, suggesting that this dysregulation is highly related to hepatocytes malignant transformation. Furthermore, high upregulation of *TBX21* was found only in BRAF V600E-overexpressed hepatocytes. TBX21 is a transcription factor responsible for developmental processes and regulation of Th1 cytokine and interferon gamma (IFNG) [68]. It was confirmed in another study that high expression of *TBX21* is related to poor prognosis of patients with breast cancer and lung adenocarcinoma [68,69]. Moreover, we found that Huh7 cells with BRAF V600E overexpression exhibited similar changes in gene expression compared with THLE-2 cells (Figure S2). Huh7 is a human hepatoma-derived cell line and according to the Cancer Cell Line Encyclopedia, 108 genes are mutated (including TP53) [70,71]. Analysis of the Huh7 genome profile revealed highly heterogeneous cell populations with a diverse number of chromosomes [72]. Nevertheless, our results suggest that the BRAF V600E mutation partially affects common pathways regardless of hepatic cell line (e.g., *MMP10*, *BMP6*, *IL1B*). Although we identified several BRAF V600E-driven gene expressions in THLE-2 cells, how these expressions are modulated by BRAF V600E-MAPK/ERK axis is still unknown. Therefore, our future studies will focus on identifying molecular mechanisms underlying aberrantly regulated gene expression by BRAF V600E-MAPK/ERK.

Most BRAF mutations have increased kinase activity, but some BRAF mutants have an opposite effect [73]. Functional analysis of kinase-impaired mutants has revealed that ERK is activated due to CRAF and BRAF heterodimerization resulting in signal transduction in a RAS-independent way [74]. It has been shown that BRAF mutations in codons 594 and 596 significantly differ from V600E in terms of molecular, pathological, and clinical features in colorectal cancer [75]. Studies using a murine melanoma model have shown that tumorigenesis is closely related to the kinase-dead BRAF (D594A) and oncogenic RAS. Therefore, it is assumed that BRAF mutations in the DFG motif cause tumor development through its enhanced interaction with RAS mutant proteins [74]. In our study, BRAF D594A did not influence the ERK phosphorylation in hepatocytes (THLE-2 cell line) by itself. This effect may be observed in the presence of oncogenic RAS. Interestingly, BRAF L537M and BRAF E648G overexpression in hepatocytes exhibited down-regulation of the MAPK/ERK signaling pathway. However, through several functional assays and gene expression analysis we did not observe any differences in cell phenotype, nor in the regulation of gene expression. Based on our findings we conclude that these mutations are not tumorigenic in liver cancer.

In conclusion, our study demonstrates that enhanced BRAF V600E expression in hepatocytes favors cancerous development. Therefore, further research is needed to explore the detailed mechanisms by which BRAF V600E-dependent pathways exert influence on the tumorigenesis of human liver cells.

## 4. Materials and Methods

### 4.1. Sequence Alignment

A multiple sequence alignment of the BRAF protein activation segment was performed for several species—*Homo sapiens* (NP_004324.2), *Mus musculus* (XP_011239439.2), *Bos taurus* (XP_024846960.1), *Danio rerio* (NP_001311445.1), and *Xenopus tropicalis* (XP_031754392.1)—using PRALINE software [76]. NCBI reference sequences are given in parentheses.

### 4.2. Cell Culture

THLE-2 cells (derived from primary normal human liver cells) were purchased from American Type Culture Collection (CRL-2706™; Manassas, VA, USA). THLE-2 cells were cultured in LHC-8 medium (Thermo Fisher Scientific, Waltham, MA, USA) supplemented with 10% fetal bovine serum (FBS; EURx, Gdańsk, Poland), 5 ng/mL epidermal growth factor (EGF; Thermo Fisher Scientific, Waltham, MA, USA), 70 ng/mL phosphoethanolamine (Sigma-Aldrich, Saint Louis, MO, USA), and 100 units/mL penicillin/streptomycin (Lonza, Basel, Switzerland). Cells were cultivated under standard conditions: 37 °C with 5% CO_2_, and humidified atmosphere in the incubator on a T-75 cm^2^ cell culture flask (Sigma-Aldrich, Saint Louis, MO, USA). To dissociate cell monolayer for the subculture, THLE-2 cells were washed with phosphate-buffered saline (PBS; Lonza, Basel, Switzerland) without calcium and magnesium, followed by treatment with trypsin 0.25%—EDTA in HBSS (Biosera, Nuaille, France)—for 4 min at 37 °C.

### 4.3. Plasmid Construction

Coding DNA sequence (CDS) for BRAF (NM_004333.5) was amplified using NG dART RT kit (EURx, Gdańsk, Poland) based on RNA extracted from THLE-2 cell. Next, BRAF CDS was amplified using a high-performance DNA polymerase (PrimeSTAR Max DNA Polymerase; TaKaRa, Shiga, Japan) according to the manufacturer’s instruction. Sequence of the primers for PCR-based cloning are presented in Table 3. PCR product was purified from a mixture of PCR reagents using NucleoSpin^®^ Gel and PCR Clean-up kit (Macherey-Nagel, Düren, Germany), according to the manufacturer’s instruction. Purified DNA insert and p3XFLAG-CMV (Sigma-Aldrich, Saint Louis, MO, USA) plasmid backbones were treated with restriction enzymes to obtain sticky ends. HindIII (recognition site on the 5′ end) and XbaI (recognition site on the 3′ end) were purchased from Thermo Fisher Scientific (Waltham, MA, USA) and used for reaction.

Next, both BRAF insert and p3XFLAG-CMV plasmid backbone were cleansed using NucleoSpin^®^ Gel and PCR Clean-up kit (Macherey-Nagel, Düren, Germany) before ligation. Anza™ T4 DNA Ligase Master Mix (Thermo Fisher Scientific, Waltham, MA, USA) was used to ligate plasmid backbone with BRAF insert according to manufacturer instruction. Bacterial transformation was performed using NEB 5-alpha competent *Escherichia coli* cells (New England BioLabs, Ipswich, MA, USA) by heat shock method. After that, bacterial cells were transferred to the tube with 1 mL (warmed to RT) of super optimal catabolite repression (SOC) medium (A&A Biotechnology, Gdynia, Poland), and grown on a shaker incubator (250 rpm) at 37 °C for 1 h. Next, 50 µL transformed cell suspension was spread onto a warm 60 mm LB agar (A&A Biotechnology, Gdynia, Poland) plate containing ampicillin (100 µg/mL; A&A Biotechnology, Gdynia, Poland) and incubated overnight at 37 °C. Single colonies from LB agar plate dropped into 2 mL of liquid LB (A&A Biotechnology, Gdynia, Poland) with 100 µg/mL ampicillin (A&A Biotechnology, Gdynia, Poland) in a 15 mL tube with loosely closed cap and incubated on 250 rpm shaker chamber at 37 °C, overnight. Plasmids were purified by NucleoSpin Plasmid QuickPure™ Kit (Macherey-Nagel, Düren, Germany) and verified by Sanger sequencing (Genomed, Warsaw, Poland) to confirm the BRAF insertion in a plasmid backbone. After that, plasmid was amplified in mid-size culture and subjected to site-directed mutagenesis (SDM) reactions. QuikChange II Site-Directed Mutagenesis Kit (Agilent Technologies, Santa Clara, CA, USA) was applied to generate BRAF mutations—D594A, V600E, L537M, and E648G—in the p3XFLAG-CMV + BRAF WT (wild type) plasmid, according to the manufacturer’s instructions. The QuikChange Primer Design tool (Agilent Technologies, Santa Clara, CA, USA) was used to design specific primers for reaction (Table 4). All BRAF mutations were confirmed by Sanger sequencing (Genomed, Warsaw, Poland) and used for further experiments.

### 4.4. Cell Transfection

THLE-2 cells were transfected with N-Terminal p3XFLAG-CMV plasmid backbone and with plasmids containing the following cloned BRAF variants: BRAF WT, BRAF V600E, BRAF D594A, BRAF L537M, and BRAF E648G. X-tremeGENE™ HP DNA Transfection Reagent (Roche, Mannheim, Germany) was used in ratio 1:1 to DNA in accordance with manufacturer instruction. Depending on the subsequent study, cells were transfected in 24-well or 6-well plates. Briefly, after 24 h from seeding, cells were rinsed with calcium- and magnesium-free PBS (Lonza, Basel, Switzerland). Next, a fresh medium without penicillin/streptomycin was added into the wells. The transfection complex was mixed well and incubated for 15 min at RT. Thereafter, the mixture was added to the cells in a dropwise manner. Following transfection, cells were placed in the incubator for 48 or 72 h depending on the experiment.

### 4.5. Western Blotting

The expression of proteins extracted from cells was determined using the Western blotting analysis. The study evaluated the impact of BRAF mutations on its kinase activity. Briefly, protein was extracted from cells seeded on 6-well plate using 200 µL/well ice-cold T-PER Tissue Protein Extraction Reagent (Thermo Fisher Scientific, Waltham, MA, USA Scientific) supplemented with cOmplete™ Protease Inhibitor Cocktail (Roche, Mannheim, Germany). The protein concentration was estimated by using Pierce™ BCA Protein Assay Kit (Thermo Fisher Scientific, Waltham, MA, USA). Equal amounts (10 µg) of each sample were loaded into the well of 4–15% Mini-PROTEAN TGX Gels (Bio-Rad, Hercules, CA, USA) along with the 3-Colour Pre-stained Protein Marker (10–245 kDa; Blirt, Gdańsk, Poland) in running buffer (25 mM Tris; 190 mM glycine; 0.1% SDS; Sigma-Aldrich, Saint Louis, MO, USA). Next, the protein was transferred from the gel to the hydrophobic, 0.45 µm pore size Immobilon-FL PVDF Membrane (Millipore, Burlington, MA, USA) by wet transfer system. The transfer buffer consisted of 25 mM Tris (Sigma-Aldrich, Saint Louis, MO, USA), 190 mM glycine (Sigma-Aldrich, Saint Louis, MO, USA), and 20% methanol (Chempur, Piekary Śląskie, Poland). The electrophoretic transfer was run at 150 mA for 1 h and 200 mA for 2 h in the case of 1 or 2 gels in the transfer tank, respectively. After that, the membrane was blocked in blocking buffer (5% skim milk in TBST) for 1 h at RT on the rocking platform shaker and incubated overnight in the primary antibody solution against the target protein in a cold room (4 °C) with gentle agitation. All antibodies (primary and secondary) used in the Western blot method were diluted in blocking buffer and are presented in Table 5. Proteins were visualized using enhanced chemiluminescence (ECL) detection reagents (Amersham ECL Western Blotting Analysis System; GE Healthcare, Chicago, IL, USA) according to the manufacturer’s instruction and captured by the ChemiDoc XRS+ System (Bio-Rad, Hercules, CA, USA). Band density of the target proteins was estimated using ImageJ software.

### 4.6. Wound Healing Assay

A wound healing assay (scratch assay) was applied to estimate changes in THLE-2 cell migration after transfection with plasmids containing BRAF mutations. THLE-2 cells were grown in complete medium on 6-well plate (2 × 10^5^ cells/well) and transfected 24 h from cell seeding. After 6 h from cell transfection, the cell monolayer was scraped using 200 µL pipet tip to obtain a scratch. The medium was aspirated to remove detached cells and debris from the wells. Then, cells were rinsed with PBS (Lonza, Basel, Switzerland) and 2 mL of fresh, complete medium was added. After 72 h, cells were washed with PBS and fixed with 4% paraformaldehyde (PFA; Sigma-Aldrich, Saint Louis, MO, USA) in PBS for 15 min at RT with gentle shaking. Next, cells were washed with PBS and stained with hematoxylin and eosin for picture acquisition. Briefly, fixed cells were immersed in hematoxylin solution (concentration 6 g/L; Sigma-Aldrich, Saint Louis, MO, USA) for 1.5 min at RT, then flushed once with distilled water to wash off excessive dye. Counterstaining was performed by eosin Y solution (concentration 0.5% in acidified ethanol; Sigma-Aldrich, Saint Louis, MO, USA) for 15 s at RT. To obtain optimal staining intensity, cells were rinsed twice in distilled water and immediately photographed under the light microscope at 4× magnification (Nikon Eclipse E200, Tokyo, Japan). The number of cells in the scratch area was calculated using ImageJ software and presented on the chart.

### 4.7. Cell Proliferation Test (WST-1 Test)

The influence of selected BRAF mutation on cell proliferation and growth was investigated using Cell Proliferation Reagent WST-1 (Roche, Mannheim, Germany). THLE-2 cells were seeded on a 24-well plate at a concentration of 5 × 10^4^ cells/well. After 24 h cells were transfected with N-Terminal p3XFLAG-CMV plasmid (Sigma-Aldrich, Saint Louis, MO, USA) or with containing the following BRAF: WT, D594A, V600E, L537M, and E648G. Cells were incubated for 48 h under standard condition. Next, the absorbance was measured with accordance to manufacturer instructions using Synergy LX multi-mode reader (BioTek, Winooski, VT, USA).

### 4.8. Cell Invasion Assay

Invasion assay was measured by counting the THLE-2 invaded cells after overexpression with empty N-Terminal p3XFLAG-CMV plasmid (Sigma-Aldrich, Saint Louis, MO, USA) or with plasmid containing BRAF WT and BRAF V600E. Corning BioCoat™ Matrigel Invasion Chamber (Corning, NY, USA) with 8 μm pores inserts was used to perform the analysis according to manufacturer instructions. After 24 h from transfections THLE-2 cells were seeded in the number of 0.5 × 10^5^ in serum-free LHC-8 medium and incubated for 24 h under standard conditions. Non-invasive cells from the upper surface of the membrane were removed with cotton swabs. The invading cells were fixed and stained using Differential Quick III Stain Kit (Polysciences, Inc., Warrington, PA, USA), and photographed under the light microscope (Nikon Eclipse E200, Tokyo, Japan). The number of cells was calculated using ImageJ software and presented as a bar chart.

### 4.9. BGI RNA-Seq (Transcriptome) Sequencing

RNA was extracted from the THLE-2 cells after 48 h from cell transfection with N-Terminal p3XFLAG-CMV plasmid (Sigma-Aldrich, Saint Louis, MO, USA) and with plasmid containing BRAF WT and BRAF V600E using RNA extraction kit NucleoSpin RNA (Macherey-Nagel, Düren, Germany). Changes in gene expression were quantified using DNB-SEQ 30M PE100 reads sequencing by BGI Genomics (Shenzhen, China). Fastq files were aligned to reference genome GRCh38 using STAR aligner [77]. Read counts were created using GENCODE v34 and featureCounts [78]. Differentially expressed transcripts were determined using DESeq2 [79] using a false discovery rate (FDR) of 0.1.

### 4.10. Network Analysis

Results obtained from BGI RNA-Seq (transcriptome) sequencing were subjected to QIAGEN Ingenuity Pathway Analysis (QIAGEN IPA) software provided by Ingenuity Systems [80] for further analysis. A *p*-value cut-off of 0.1 after Benjamini–Hochberg multiple-testing correction was applied for RNA-Seq data. To identify the interaction between significant DEGs, Cytoscape 3.8.2 (ClueGO 2.5.7) software [81] was applied.

### 4.11. cDNA Synthesis

Total RNA isolated from THLE-2 cells was used as a template to synthesize cDNA by NG dART RT kit (EURx, Gdańsk, Poland) in accordance with the manufacturer instruction. The tubes were incubated in GeneAmp^®^ PCR System 9700 thermal cycler (Applied Biosystems, Waltham, MA, USA) at 50 °C for 60 min, followed by 85 °C for 5 min, and cooled down to 4 °C.

### 4.12. Real-Time PCR (qPCR)

Results obtained from (transcriptome) sequencing were validated by real-time PCR for selected genes using RT HS-PCR Mix SYBR^®^ C (A&A Biotechnology, Gdynia, Poland) according to manufacture instruction. The sequences of the primers (purchased from Sigma-Aldrich, Saint Louis, MO, USA) are indicated in Table 6. Reactions were performed using the LightCycler^®^ 96 Instrument (Roche, Mannheim, Germany) and analyzed with LightCycler 96 SW 1.1 (Roche, Mannheim, Germany) software. PCR product was melted after amplification to confirm its specificity.

### 4.13. Statistical Analysis

Statistical analysis of the data was examined using GraphPad PRISM software version 6 (GraphPad Software, La Jolla, CA, USA). The effect of single mutations was analyzed using a one-way analysis of variance (ANOVA), with Bonferroni correction. The level of statistical significance was considered as: * *p* < 0.05; ** *p* < 0.01; *** *p* < 0.001. Data were presented as a bar graph based on means with ±SEM (standard error of the mean) or ±SD (standard deviation) and drawn using GraphPad PRISM software (GraphPad Software, La Jolla, CA, USA).

## Figures and Tables

**Table 1:** BRAF mutations were identified by ICGC and subjected to functional analysis.

Mutation	Mutation ID	Genomic DNA Change	Type	Projects Mutation Observed	Country
L537M	MU3683890	chr7:g.140476797 A > T	Single base substitution leading to a missense mutation	LIRI-JP:1/258	Japan
D594A	MU30632423	chr7:g.140453154 T > G		LIRI-JP:1/258	Japan
V600E	MU62030	chr7:g.140453136 A > T		LICA-FR:1/240	France
E648G	MU1115756	chr7:g.140449136 T > C		LIRI-JP:1/258	Japan

**Table 2:** The 25 most strongly up-regulated and down-regulated genes in THLE-2 cells. Results are presented as a fold change (log ratio) in BRAF V600E versus BRAF WT comparisons.

Up-Regulated		Down-Regulated	
BRAF V600E vs. BRAF WT			
Gene	Fold Change (Log Ratio)	Gene	Fold Change (Log Ratio)
*MMP10*	4.847	*CXCL10*	−0.948
*TBX21*	4.205	*RSAD2*	−0.855
*SERPIND1*	2.823	*CXCL11*	−0.675
*COL5A3*	2.600	*CMPK2*	−0.619
*ADAMTS18*	2.338	*CCL5*	−0.557
*IL33*	2.325	*HSH2D*	−0.500
*BMP6*	2.185	*NT5C3A*	−0.488
*NTSR1*	2.173	*IFIT2*	−0.449
*LRRC15*	1.903	*BATF2*	−0.435
*TAGLN3*	1.859	*RTP4*	−0.435
*ESM1*	1.834	*IFIT1*	−0.429
*MMP9*	1.809	*IFIT3*	−0.413
*SIGLEC15*	1.741	*USP18*	−0.405
*ITGAX*	1.716	*GBP4*	−0.400
*TRPV3*	1.681	*GMPR*	−0.400
*DIRAS3*	1.625	*SLC15A3*	−0.391
*MMP1*	1.569	*C4orf33*	−0.381
*IL13RA2*	1.545	*LGALS9*	−0.377
*MMP3*	1.493	*TNFSF10*	−0.376
*RCSD1*	1.464	*OASL*	−0.373
*TNFSF15*	1.447	*BST2*	−0.371
*NOX5*	1.445	*GBP1*	−0.362
*CHRNA9*	1.287	*MX2*	−0.358
*EGR3*	1.250	*PSMB9*	−0.352
*IL1B*	1.233	*HERC5*	−0.342

**Table 3:** List of primers used for PCR amplification of DNA insert for BRAF. Primers sequence consist of hybridization sequence that binds to the CDS sequence (marked in black), restriction site sequence (marked in red), that was specific for selected restriction enzymes, and leader sequence, which was extended by additional bases on the 5′ end of the primer to improve cutting efficiency (marked in blue).

Primer Name	Primer Sequence	Amplicon Size
HindIII_18	Forward: GGAAGCTTGCGGCGCTGAGCGGTGGC	2298 bp
XbaI_20	Reverse:GGTCTAGATCAGTGGACAGGAAACGCAC	

**Table 4:** Sequences of the primers used for site-directed mutagenesis.

Mutation	Position	Primer Sequences		Length (nt.)
D594A	a1781c	F R	5′-TCACTGTAGCTAGACCAAAAGCACCTATTTTTACTGTGAGG-3′ 5′-CCTCACAGTAAAAATAGGTGCTTTTGGTCTAGCTACAGTGA-3′	41
L537M	t1609a	F R	5′-GGAGATGGTGATACATGCTGGAGCCCTCACA-3′ 5′-TGTGAGGGCTCCAGCATGTATCACCATCTCC-3′	31
V600E	t1799a	F R	5′-CCACTCCATCGAGATTTCTCTGTAGCTAGACCAAAAT-3′ 5′-ATTTTGGTCTAGCTACAGAGAAATCTCGATGGAGTGG-3′	37
E648G	a1943g	F R	5′-GGTAACTGTCCAGTCATCAATCCATACAGAACAATTCCAAATG-3′ 5′-CATTTGGAATTGTTCTGTATGGATTGATGACTGGACAGTTACC-3′	43

**Table 5:** List of primary and secondary antibodies used for Western blotting.

Primary Antibody					Secondary Antibody	
Antibody	Dilution	Host	Molecular Weight (kDa)	Supplier	Antibody	Dilution
Anti-Flag M2	1:2000	mouse, monoclonal	94	Sigma-Aldrich	Anti-Mouse IgG (whole molecule)–Peroxidase antibody produced in goat	1:5000
ERK1/2 (C-9)	1:1000	mouse, monoclonal	44/42	Santa Cruz Biotechnology	Anti-Mouse IgG (whole molecule)–Peroxidase antibody produced in goat	1:5000
Phospho-p44/42 MAPK (Erk1/2)	1:1000	rabbit, monoclonal	44/42	Cell Signaling Technology	Anti-Rabbit IgG (whole molecule)–Peroxidase antibody produced in goat	1:5000
β-Actin (13E5)	1:1000	rabbit, monoclonal	45	Cell Signaling Technology	Anti-Rabbit IgG (whole molecule)–Peroxidase antibody produced in goat	1:5000

**Table 6:** Sequence of primers used for PCR.

Gene	GenBank Acc. No.	Primer Sequence	Length	Exon Boundary
*BRAF*	NM_004333	F: CCCCAAGTCACCACAAAAACC R: CGGACTGTAACTCCACACCTT	90	3–4
*BMP6*	NM_001718	F: AAGAAGGCTGGCTGGAATTT R: GAAGGGCTGCTTGTCGTAAG	170	3–4
*CXCL11*	NM_005409	F: TCGAAGCAAGCAAGGCTTAT R: GTCCTTTCACCCACCTTTCA	221	2–3
*IL1B*	NM_000576	F: TCCAGGGACAGGATATGGAG R: TCTTTCAACACGCAGGACAG	133	5–6
*TBX21*	NM_013351	F: CCGTGACTGCCTACCAGAAT R: ATCTCCCCCAAGGAATTGAC	158	4–6
*RSAD2*	NM_080657	F: CTCGCCAGTGCAACTACAAA R: CACCAACTTGCCCAGGTATT	182	1–2
*MMP10*	NM_002425	F: GTGGAGTTCCTGACGTTGGT R: AGCCTGGAGAATGTGAGTGG	181	2–3
*SERPIND1*	NM_000185	F: GAAGTTGATGGGGATCAGGA R: GTCGACAGTGAAGCGGACTT	190	4–5
*B2M*	NM_004048	F: GAGGCTATCCAGCGTACTCCA R: CGGCAGGCATACTCATCTTTT	248	1–2
*GAPDH*	NM_001256799	F: GGAGCGAGATCCCTCCAAAAT R: GGCTGTTGTCATACTTCTCATGG	197	4–6

## Data Availability

RNA-Seq Data is available on GEO, accession number: GSE194425.

## Supplementary Materials

The following supporting information can be downloaded at: https://www.mdpi.com/article/10.3390/ijms23031548/s1.
