# Supplementary material for: Oncogenic Mutation BRAF V600E Changes Phenotypic Behavior of THLE-2 Liver Cells through Alteration of Gene Expression
Source: Int J Mol Sci. 2022 Jan 28;23(3):1548. doi: 10.3390/ijms23031548 (PMC8836259; doi:10.3390/ijms23031548)
Supplement: Supplementary file 1 [file ijms-23-01548-s001.zip › ijms-1498182-supplementary.pdf]

International Journal of
Molecular Sciences

Supplementary Materials

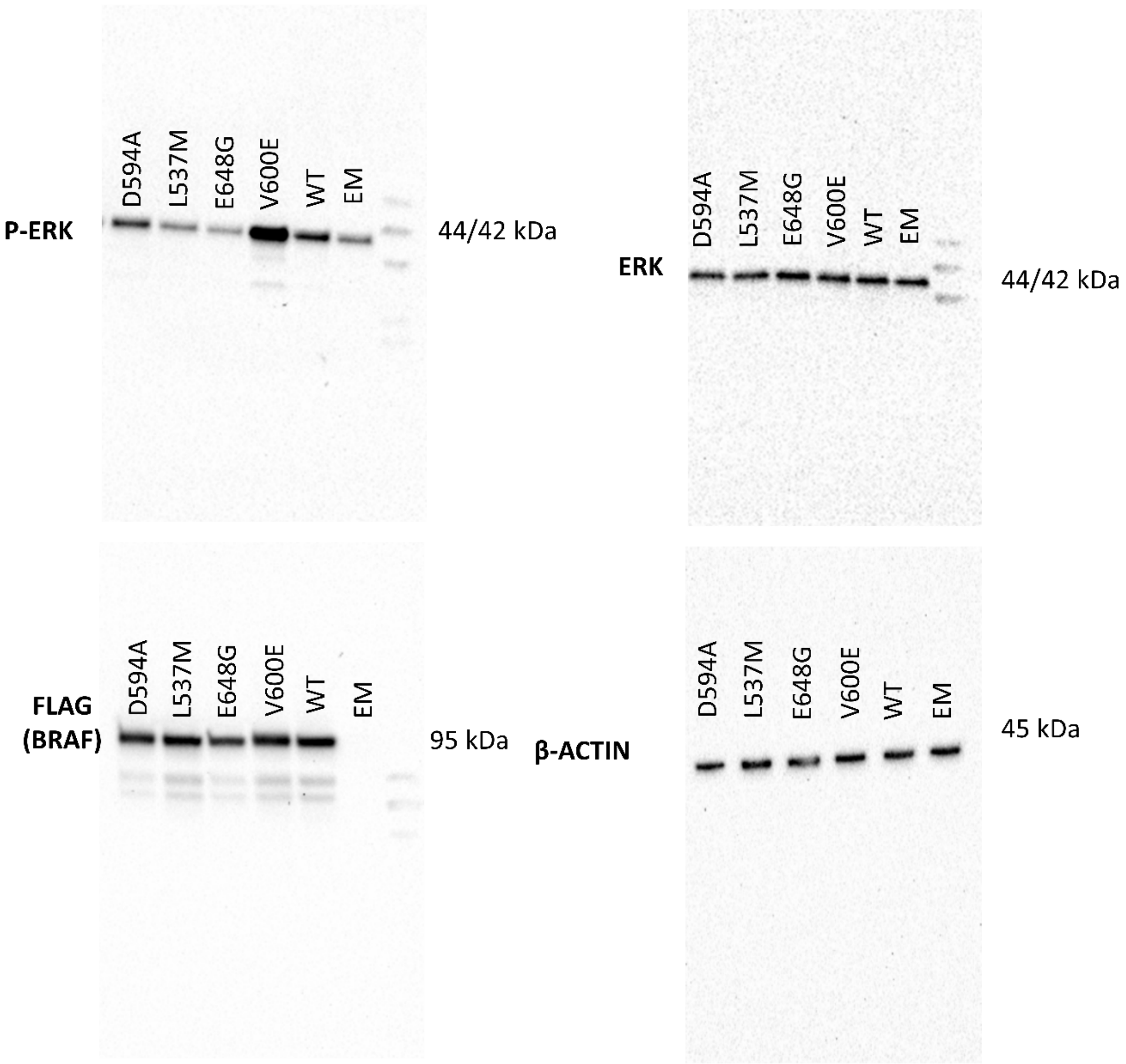

Figure S1. Uncropped pictures of Western blot for P-ERK, FLAG (BRAF), ERK, and β-ACTIN protein expression in THLE-2 cells. THLE-2 cells were overexpressed with p3XFLAG-CMV empty plasmid (EM), plasmid containing BRAF WT (WT) or BRAF mutations (V600E, E648G, L537M, D594A).

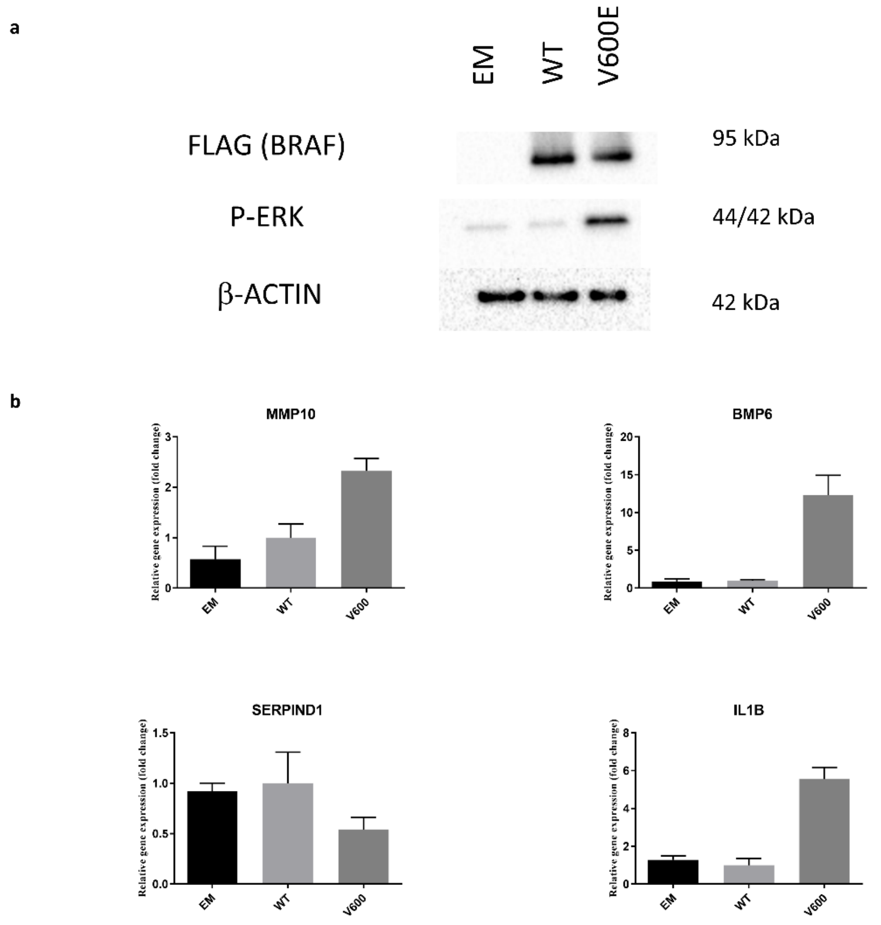

Figure S2. Influence of BRAF V600E mutation on the selected gene expression in Huh7 cell line. (**a**) Expression of FLAG (BRAF), ERK-phosphorylated (P-ERK), and β-ACTIN in Huh7 cells overexpressed with p3XFLAG-CMV empty plasmid (EM), or plasmid containing BRAF WT (WT) and BRAF mutation (V600E). (**b**) Real-time qPCR results of deregulated gene expression in Huh7 cells with BRAF V600E overexpression. Data are presented as a fold change in contrast to the expression of BRAF WT transfected cells (± SEM). B2M was used as a reference gene, n=2.

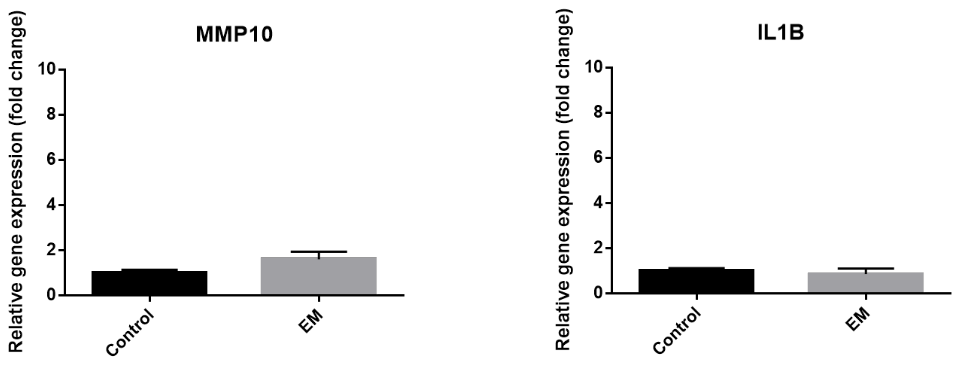

Figure S3. Influence of DNA transfection on the selected gene expression in THLE-2 cell line. Real-time qPCR results of selected gene expression in THLE-2 cells with Empty plasmid (EM) overexpression. Data are presented as a fold change in contrast to the expression of non-transfected cells (Control) (± SEM). B2M was used as a reference gene, n=3.
